# Changes in bursal B cells in chicken during embryonic development and early life after hatching

**DOI:** 10.1038/s41598-018-34897-4

**Published:** 2018-11-15

**Authors:** Kwang Hyun Ko, In Kyu Lee, Girak Kim, Min Jeong Gu, Hyun Young Kim, Byung-Chul Park, Tae Sub Park, Seung Hyun Han, Cheol-Heui Yun

**Affiliations:** 1 Department of Agricultural Biotechnology and Research Institute for Agriculture and Life Sciences, Seoul National University, Seoul, 08826 Republic of Korea; 2 Biomodulation Major and Center for Food Bioconvergence, Seoul National University, Seoul, 08826 Republic of Korea; 3 Department of Oral Microbiology and Immunology, Dental Research Institute, and Brain Korea 21 Plus Program, School of Dentistry, Seoul National University, Seoul, 08826 Republic of Korea; 4 Institute of Green Bio Science Technology, Seoul National University, Pyeongchang, 25354 Republic of Korea

## Abstract

The bursa of Fabricius, the primary lymphoid organ for B cell development found only in birds, offers novel approaches to study B cell differentiation at various developmental stages. Here, we explored the changes and mechanism involved in the developmental stages of bursal B cells. The bursal B cells rapidly increased in the late embryonic stage and around hatching, which coincided with changes in specific cell surface markers. Moreover, the cells in the bursa were divided by size into small (low forward- and side-scatter) or large (high forward- and side-scatter) via flow cytometry. It is intriguing that the proportion of small and large B cells was reversed during this period. Because little is known about this phenomenon, we hypothesized that size-based B cell population could be used as an indicator to distinguish their status and stage during B cell development in chicken. The results demonstrated that large B cells are actively proliferating cells than small B cells. Additionally, large B cells showed higher mRNA expression of both proliferation- and differentiation-associated genes compared to small B cells. Taken together, these data show that large bursal B cells are the main source of proliferation and differentiation during B cell development in chickens.

## Introduction

B cell development in chickens occurs in a primary lymphoid organ unique to birds, the bursa of Fabricius, which provides a useful experimental model to study the early stages of B cell differentiation^1–4^. The bursa develops from the epithelial rudiment of the cloaca around embryonic day (E) 4–5 and is colonized by pre-bursal cells from the hematopoiesis site between E8 and E14^3,5^. Following the migration and colonization of the cells into bursal follicles while immunoglobulin (Ig) gene rearranged, the cells undergo rapid expansion^4,6^. The bursal cells continuously divide, reaching maximum size at 8–10 weeks of age, and then gradually go through atrophy^4^. Unlike mammals, in which the developmental stages of B cells are divided into pro-B cells, pre-B cells and immature B cells^7^, chickens have three distinct B cell stages: pre-bursal, bursal and post-bursal B cells^8^. It is hard to separate the stages of chicken B cells into categories analogous to mammalian B cells because there are very few surface markers for identifying the differentiation stage of chicken B cells. Moreover, in contrast to mice, gene-targeting approaches are extremely limited in chicken studies, especially for B cell development.

There are several factors that regulate B cell development in chickens. *Aiolos*, *Ikaros*, and *E2A* are expressed early in the embryonic bursa and have critical roles in the expansion and maturation of bursal B cells^9^. In addition to regulatory functions of various molecules, many signaling pathways are also involved in the differentiation of chicken B cells. Recently, transcriptional analysis has revealed that the MAPK, Wnt, Notch and JAK-STAT signaling pathways are essential in B cell development and that those pathway-related genes are differentially expressed in bursal B cells at various stages of B cell development^8^.

A few strategies exist for distinguishing the developmental stages of B cells. In both humans and mice, cluster of differentiation (CD) proteins are useful markers to classify different stages of B cell development. Additionally, the differentiation stage of B cells is defined by expression of surface immunoglobulin (Ig) and rearrangements of Ig H-chain (IgH) and Ig L-chain (IgL) genes. However, chicken B cells undergo IgH and IgL gene rearrangement at the same time during B cell development, indicating that Ig chain rearrangement could not be a differentiation marker of B cell stages in chickens. Cell size can also be an indicator to distinguish B cell stages in humans and mice. For example, mammalian pre-B cells are separated into 2 groups based on cell size (large and small pre-B cells). Large pre-B cells undergo a large number of cell proliferation, which produce μ chains, and then they differentiate into non-proliferative small pre-B cells. However, B cell development studies based on cell size in chickens have not been reported previously.

For bursal B cell development in chickens, changes in immunoglobulin and involvement of gut antigens are suggested as key factors^2,10,11^. Recently, the identification of gene expression related with immunological functions in chicken intestinal epithelial lymphocytes has been studied^12^ and B cell development was examined using RNA sequencing analysis to find differential gene expression in the different developmental stages^8^. However, those molecular changes and mRNA expression did not provide indicators to distinguish B cell status and stages in the chicken.

In the present study, we hypothesized that a new strategy for the classification of B cells based on cell-size and granularity could be useful to distinguish their status and stage during B cell development in chicken. We show that in the bursa, large B cells were both more proliferative and differentiated than small B cells. Furthermore, the ratio of small to large B cells reverses as the chicken grows, suggesting a change in the status and stage of the B cells.

## Results

### Bursal B cells undergo rapid expansion during the late embryonic stage in chickens

**Figure 1:**
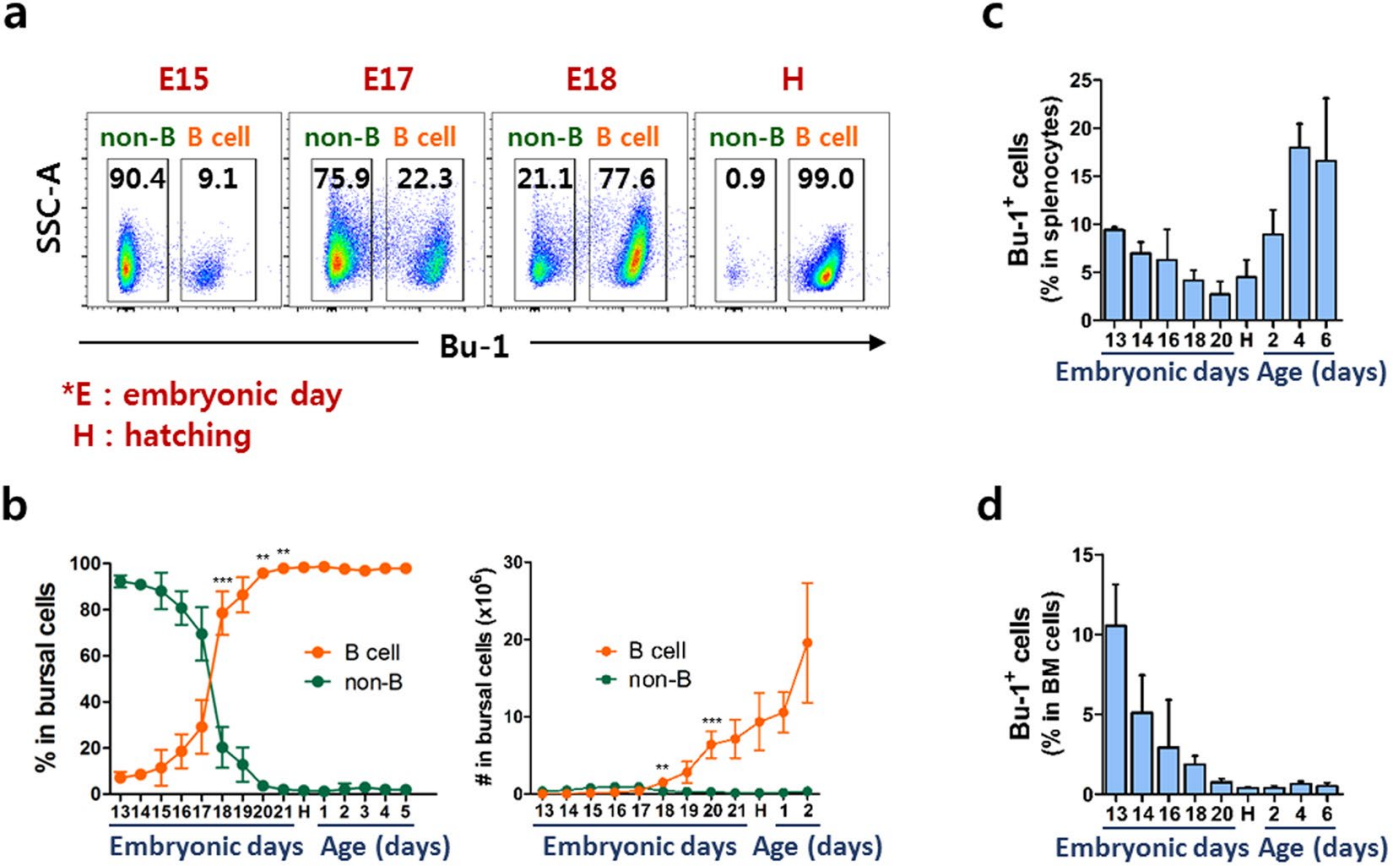
Bursal B cells rapidly increased at the late embryonic stage in chickens. Bursal cells were taken from chicks aged E13 to 2–6 days old and stained with anti-Bu-1 antibody, which were regarded as bursal B cells. (**a**) Changes in bursal B cell population at E15, E17, E18, and hatching (H). Numbers in each area indicate the percentage of non-B or B cells in the bursa. (**b**) The percentage and absolute number of bursal B cells during embryonic and post-hatching stages. Each data point represents the mean value of four to ten chickens. The frequency of B cells in comparison to non-B cells from the spleen (**c**) and bone marrow (**d**) at the indicated stage. More than 5 samples per embryonic stage were analyzed, and all results represent at least two independent experiments. Each data point represents the mean of four to ten chickens with SD. **P < 0.01, ***P < 0.001.

Pre-bursal B cells from the dorsal mesenchyme, embryonic spleen, and bone marrow (BM) colonize in the bursa of Fabricius during embryonic development between embryonic day (E)8 and E14 and undergo rapid B cell proliferation^5,9^. We used CD45 and Bu-1 antibodies to analyze chicken B cells. CD45 was expressed on almost all bursal cells whereas Bu-1 was expressed on B cell lineages in the bursa of Fabricius (Supplementary Fig. S1a–d). To explore changes in the proportion of bursal B cells, we assessed the expression of Bu-1 during the developmental stages. Interestingly, bursal B cells substantially increased in the late embryonic stage, especially at E17–18, suggesting that it is a critical time point for the development and homeostatic proliferation-like expansion of B cells in the bursa of Fabricius (Fig. 1a,b, Supplementary Fig. S2) to overcome lymphopenic condition. Splenic B cells gradually decreased until hatching then increased thereafter, indicating that B cells emigrate from the bursa to the spleen after hatching (Fig. 1c). B cell progenitors in the BM gradually disappeared and almost all, if not entire, cells were no longer present around hatching (Fig. 1d).

### Bursal B cells go through various phenotypic changes during embryonic development

**Figure 2:**
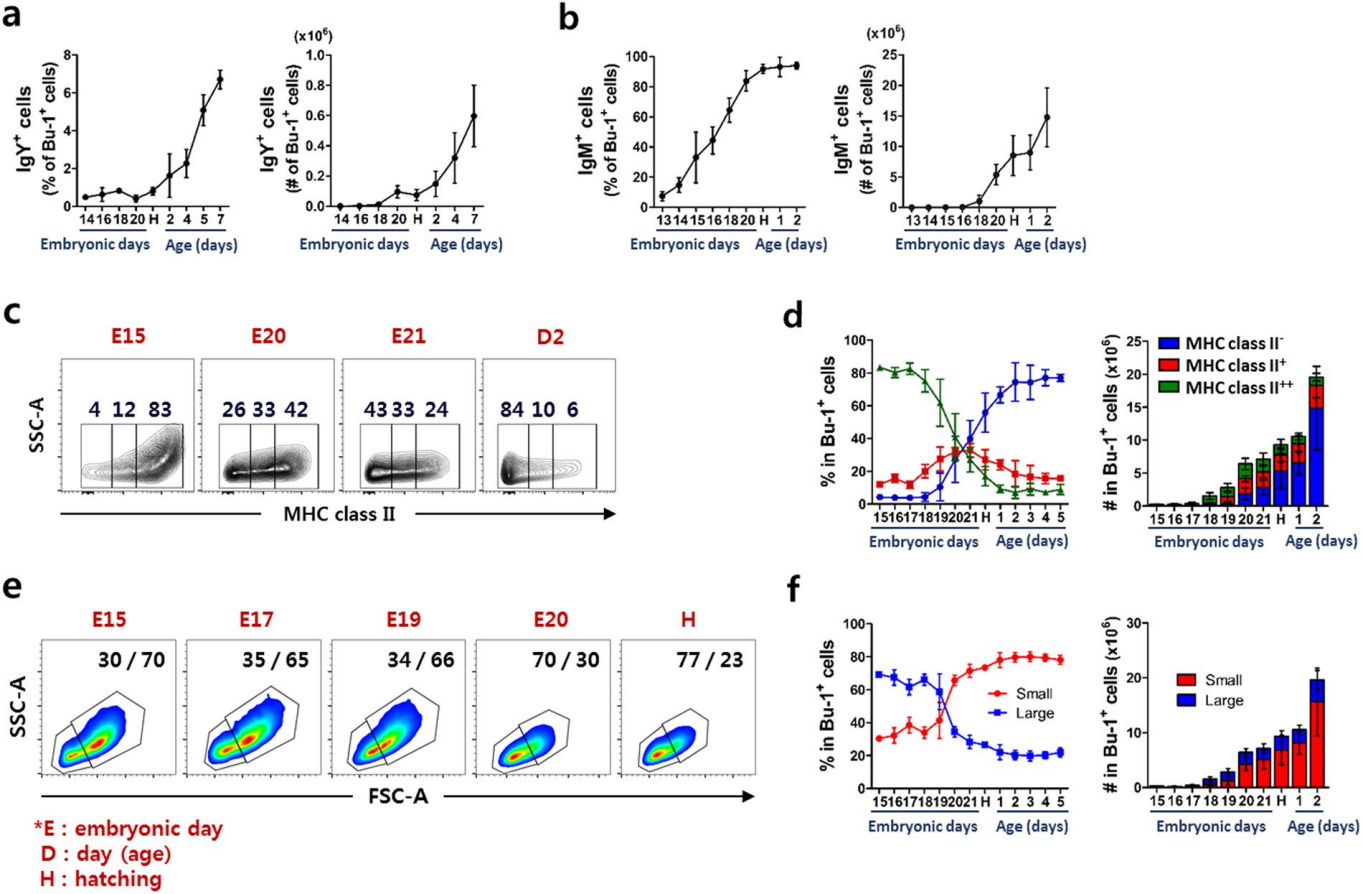
The phenotype of bursal B cells rapidly changes at around hatching. Single cells produced from the bursa were stained with anti-Bu-1 antibody together with anti-IgY, -IgM or -MHC class II antibody. The percentage and absolute number of (**a**) IgY^+^ and (**b**) IgM^+^ bursal B cells before and after hatching was examined using flow cytometry. (**c**,**d**) Surface expression of MHC class II on bursal B cells was classified as a negative (−), low (+) and high (++). (**c**) Expression of MHC class II in bursal B cells at indicated stage. The number in each area indicates the percentage of cells. (**d**) The percentage and absolute number of MHC class II expression in bursal B cells during the embryonic and post-hatching stages. (**e**,**f**) B cells were distinguished by cell size as small (low forward-scatter) or large (high forward-scatter) using flow cytometry. (**e**) Differential display of small or large bursal B cells at indicated stage. Numbers in each area show the percentage of small or large cells. (**f**) The percentage and absolute number of small or large bursal B cells during the embryonic and post-hatching stages. Each data point represents the mean of four to ten chickens with SD.

The acquisition of phenotypic characteristics, such as surface molecules, coincides with B cell development in chickens^6,13^. To determine which B cell subsets are associated with rapid expansion, we analyzed the expression of surface IgY, IgM, and IgA on the bursal B cells at various developmental stages. The expression of surface IgY on the bursal B cells was undetectable at hatching. However, the percentage and absolute number of IgY^+^ bursal B cells significantly increased after hatching (Fig. 2a). IgM^+^ B cells in the bursa were detectable at around E13, after which both the proportion and absolute number of the cells gradually increased until hatching (Fig. 2b). The number of IgA^+^ B cells in the bursa was extremely low, at less than 1% in all stages examined (E13–2 days old, data not shown).

MHC class II expression during early B cell development is essential for the maturation of murine B cells^14,15^. To explore whether MHC class II expression is relevant to the developmental stage of B cells, we classified MHC class II expression as either negative (−), low (+), or high (++) by using flow cytometry analysis. At the earliest stage examined (E15), almost all bursal B cells expressed surface MHC class II at a high level. The expression rapidly decreased (Fig. 2c), and the proportion of high-expressing cells changed from 41 to 27% on E20 and E21, respectively, and the proportion of negative and positive cells was reversed at hatching (Fig. 2d).

To further examine the morphological characteristics, we distinguished bursal B cell populations based on the cell size and granularity as either small (low forward- and side-scatter) or large (high forward- and side-scatter) (Supplementary Fig. S4). The small and large B cell populations were clearly separated with a higher proportion of large B cells than small B cells during the earliest embryonic stages examined (E15–17). Interestingly, the proportion rapidly reversed at E19–20 and small B cells became the main population, suggesting that the proportional changes are an indicator of B cell status during the development stages (Fig. 2e,f). Because little is known about this change, we further investigated the relationship between cell size and B cell development in chicken bursa.

### Large B cells are actively proliferating cells

**Figure 3:**
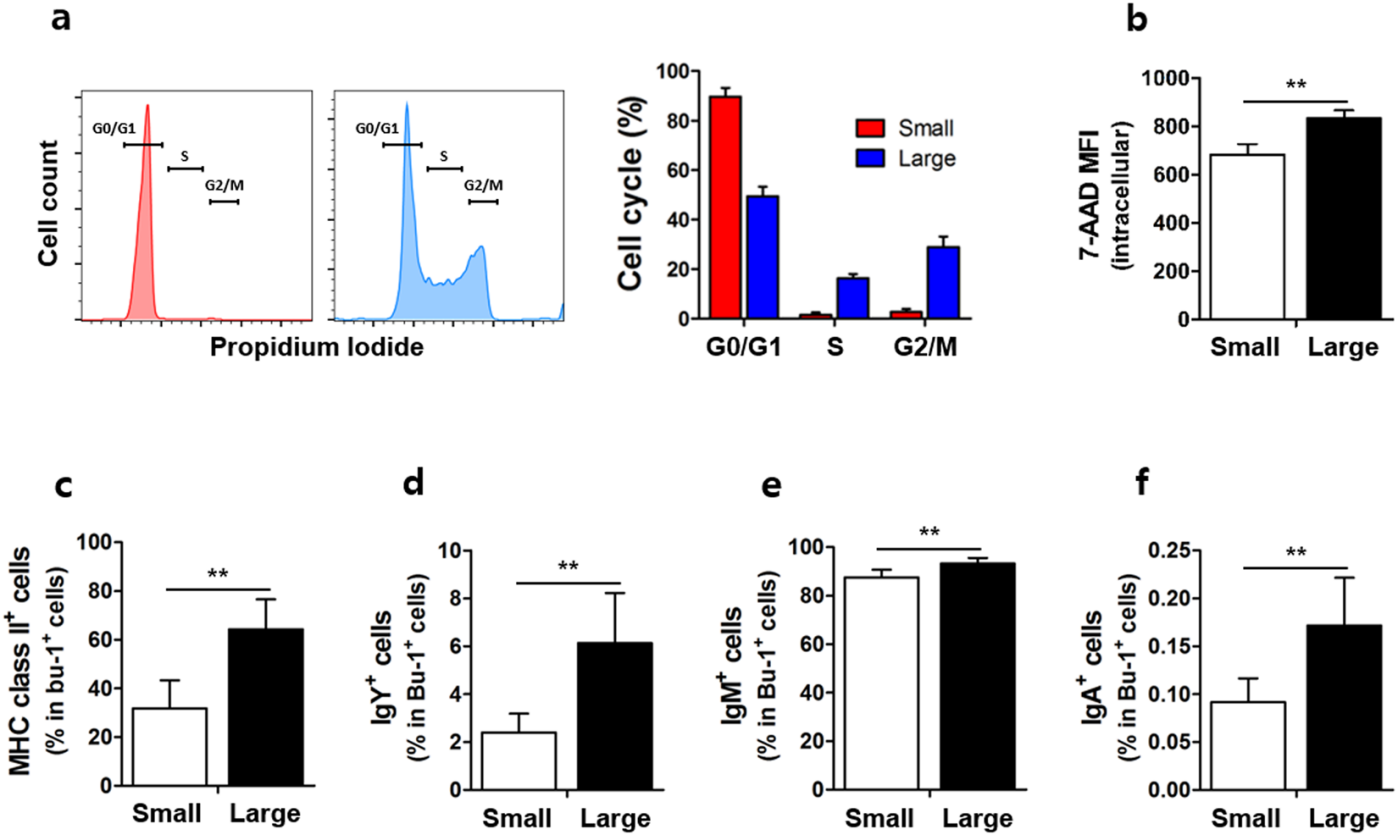
Large B cells are proliferating cells with distinct surface marker expression. Single cells were produced from the bursa of (**a**,**b**) E17 or (**c**–**f**) 2-week-old chicks. Cell cycle and DNA content were assessed using propidium iodide (PI) and 7-amino-actinomycin D (7-AAD) intracellular staining, respectively. (**a**) The percentage of G0/G1, S, and G2/M phase and (**b**) 7-AAD expression in small and large B cells as mean fluorescence intensity (MFI) are shown. The cell surface expression of (**c**) MHC class II, (**d**) IgY, (**e**) IgM, and (**f**) IgA in small and large B cells from 2-week-old chicks. **P < 0.01, ***P < 0.001.

To discern whether small or large B cells are associated with rapid proliferation of bursal B cells at each developmental stage, we analyzed the cell cycle of small and large B cells by analysis using propidium iodide (PI) staining. The proportion of S and G2/M phase was much higher in large B cells than in small B cells while the ratio of G0 resting phase is higher in small cells, indicating that large B cells were actively proliferating (Fig. 3a). 7-AAD intracellular staining showed that total DNA content was significantly higher in large B cells than in small B cells (Fig. 3b). Furthermore, we examined surface immunoglobulin and MHC class II expression on small and large B cells. The expression of surface MHC class II (Fig. 3c) was significantly higher in the large B cells than in the small B cells. Additionally, large B cells had a significantly higher expression of IgY (Fig. 3d), IgM (Fig. 3e), and IgA (Fig. 3f) than small cells. Collectively, these data indicate that large B cells are actively proliferating cells.

### Large B cells are responsible for rapid proliferation and differentiation during bursal B cell development in chickens

**Figure 4:**
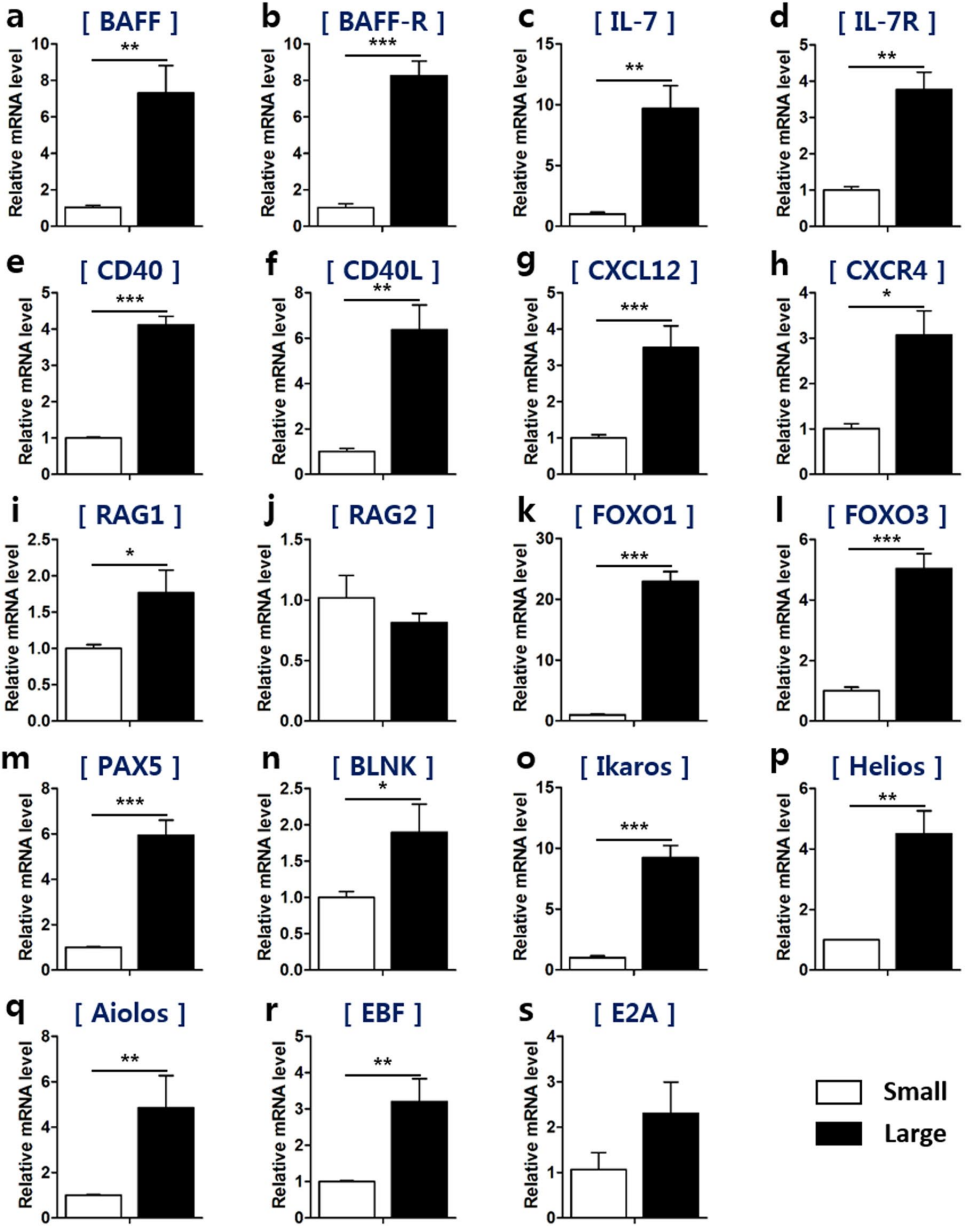
Large B cells show enhanced expression of genes for both proliferation and differentiation when compared to those of small B cells. Single cells were produced from the bursa at E17 and small and large B cells were sorted using a cell sorter. (**a**–**h**) The mRNA levels of genes associated with proliferation and colonization of B cells in small and large B cells were evaluated using qRT-PCR as follows: (**a**) *BAFF*, (**b**) *BAFF* receptor, (**c**) *IL-7*, (**d**) *IL-7* receptor, (**e**) *CD40*, (**f**) *CD40* ligand, (**g**) *CXCL12*, and (**h**) *CXCR4*. (**i**–**s**) qRT-PCR was used to determine the mRNA levels of genes associated with B cell differentiation in small and large B cells as follows: (**i**) *RAG1*, (**j**) *RAG2*, (**k**) *FOXO1*, (**l)** *FOXO3*, (**m**) *PAX5*, (**n**) *BLNK*, (**o**) *Ikaros*, (**p**) *Helios*, (**q**) *Aiolos*, (**r**) *EBF*, and (**s**) *E2A*. *P < 0.05, **P < 0.01, ***P < 0.001.

To examine whether the size-based B cell populations have different expression of B cell proliferation-related genes, we sorted bursal cells into small and large B cells and analyzed their mRNA expression. We assessed expression of both *B cell activating factor (BAFF*) and the *BAFF receptor (BAFF-R)*, which are the representative factors for the proliferation and survival signals in bursal B cells^16,17^, and found that they were higher in large B cells (Fig. 4a,b). In mammals, interleukin (IL)-7 signaling is very important in the survival, proliferation and differentiation of B cells during early B cell development^7,18^. The expression of *IL-7* and its receptor was higher in large B cells than in small B cells (Fig. 4c,d), suggesting that large bursal B cells would undergo the strong proliferation likely via the expression of these genes during embryonic development. The mRNA expression of *CD40* and *CD40 ligand*, which induce cell proliferation in B cells, was also higher in large B cells (Fig. 4e,f). The expression of the chemokine *CXCL12* and its receptor *CXCR4*, which play a role in bursal colonization, was significantly higher in large B cells than in small B cells (Fig. 4g,h). These results demonstrate that large B cells are responsible for a rapid proliferation during bursal B cell development in chickens.

In mammals, large pre-B cells undergo proliferation, but not differentiation, whereas small pre-B cells are non-proliferative and undergo V-J joining of the light chain^7,19^. Their size can be used as an indicator to distinguish their status and stage. To explore whether small and large B cells could indicate different aspects of differentiation in chickens, we assessed mRNA expression of differentiation-related genes in small and large B cells. We examined the expression of *recombination activating gene (RAG)*, which is important in inducing Ig gene rearrangement in the chicken bursa. *RAG1* mRNA expression was higher in large B cells (Fig. 4i), whereas *RAG2* mRNA levels were comparable between small and large B cells (Fig. 4j). B cell differentiation is regulated by various transcription factors^20^. Large B cells showed higher mRNA levels of the transcription factors *Forkhead box (FOXO)1*, *FOXO3*, and *Paired box 5 (PAX*5) relative to small B cells (Fig. 4k–m). Consistently, the mRNA expression of *B cell linker (BLNK)*, the downstream molecule of FOXO1-PAX5, was markedly enhanced in large B cells (Fig. 4n). The *Ikaros* family are expressed at the early stage of B cells in chickens^9^. Both *Ikaros* and *Helios* mRNA expression were higher in large B cells (Fig. 4o,p). *Aiolos* mRNA levels were comparable between small and large B cells (Fig. 4q). We also determined whether *early B cell factor (EBF)* and *E2A* showed differing expression between small and large B cells. Higher levels of both *EBF* and *E2A* mRNA expression were observed in large B cells compared with small B cells (Fig. 4r,s). Taken together, these results demonstrate that large B cells have a higher capability for proliferation and differentiation compared with small B cells during the development of the bursa of Fabricius in chickens.

### Large B cells are the major source of proliferation and differentiation

**Figure 5:**
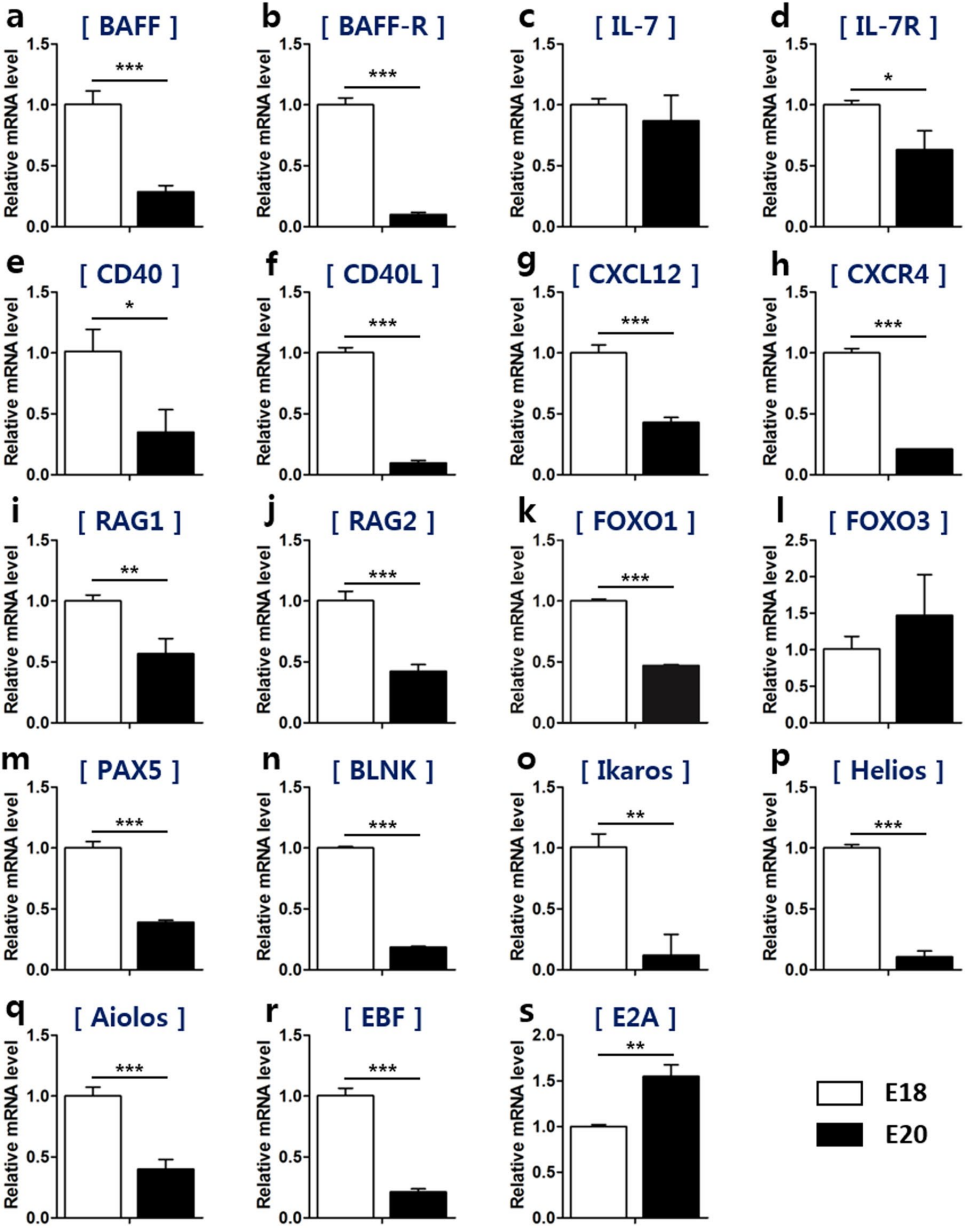
Large B cells are the main source of proliferation and differentiation in the embryonic stage. To explore whether the proportion of small and large B cells affects the status of B cells during the development, we chose two stages where the ratio of small and large B cells are dramatically reversed. Cells in the large B cell dominant stage (E18) and a small B cell dominant stage (E20) were sorted using Bu-1 positive selection, and then total RNA was extracted. (**a**–**h**) The mRNA expression of B cell proliferation- and colonization-associated genes in small and large B cells was evaluated using qRT-PCR as follows: (**a**) *BAFF*, (**b**) *BAFF* receptor, (**c**) *IL-7*, (**d**) *IL-7* receptor, (**e**) *CD40*, (**f**) *CD40* ligand, (**g**) *CXCL12*, and (**h**) *CXCR4*. (**i**–**s**) The mRNA levels of B cell differentiation-associated genes in small and large B cells were examined using qRT-PCR as follows: (**i**) *RAG1*, (**j**) *RAG2*, (**k**) *FOXO1*, (**l**) *FOXO3*, (**m**) *PAX5*, (**n**) *BLNK*, (**o**) *Ikaros*, (**p**) *Helios*, (**q)** *Aiolos*, (**r**) *EBF*, and (**s**) *E2A*. *P < 0.05, **P < 0.01, ***P < 0.001.

As shown in Fig. 2f, the proportion of small and large B cells rapidly reversed on E19–20. Given the function of small and large B cells in the bursa, we hypothesized that the proportion of small and large B cells would correlate to the status of B cells in regard to their proliferation and differentiation. To explore this, bursal B cells from E18 and E20, the time points that clearly show a different proportion of small and large B cells, were sorted and compared in regard to the expression of various genes. Large B cell-dominant E18 showed higher mRNA levels of the proliferation-related genes *BAFF*, *BAFF-R*, *IL-7*, *IL-7R*, *CD40*, and *CD40L* compared with small B cell-dominant E20 (Fig. 5a–f). The expression of *CXCL12* and its receptor *CXCR4* were also higher at E18 (Fig. 5g,h).

Consistent with the developmental function of large B cells in the bursa, the mRNA expression of the B cell differentiation-related genes *RAG1* (Fig. 5i) and *RAG2* (Fig. 5j), the transcription factors *FOXO1* (Fig. 5k) and *PAX5* (Fig. 5m), and FOXO1-PAX5’s downstream factor *BLNK* (Fig. 5n) were higher in E18 B cells compared with E20 B cells, even though *FOXO3* (Fig. 5l) was higher at E20. Furthermore, E18 B cells showed higher levels of *Ikaros* (Fig. 5o), *Helios* (Fig. 5p), and *Aiolos* (Fig. 5q) mRNA than those of E20 B cells. Additionally, *EBF*, the transcriptional regulator involved at the early B cell developmental stage, was also higher in E18 B cells than in E20 B cells, whereas *E2A* mRNA expression was lower in E18 B cells (Fig. 5r,s). Taken together, these data suggest that large B cells are the main source responsible for rapid proliferation and differentiation during chicken B cell development.

## Discussion

In 1956, it was first reported that bursa-dependent immune cells (B cells) are solely responsible for antibody production^21,22^. It has become obvious that the bursa of Fabricius is the gut-associated immune organ that provides the milieu for B cell differentiation and antibody-repertoire diversity in avian species. Compared with bone marrow, the major organ for B cell development in primates and rodents, the chicken bursa is a useful model to study B cell differentiation and function because not only it is the unique site for B cell development which is not found in other species but also is easy to anatomically access at various developmental chicken B cells^2^. There are limited developmental analogs between chicken and mammalian B cells because in chickens, not only are there few available surface markers, but the IgH and IgL chain also rearrange simultaneously^1^. Moreover, there is a pre-B cell stage in mammals, but not in chicken, during B cell development. It is important to note that studies on the developmental stages in chicken B cells are very limited, partially because chickens do not have pre-B cell stage. In the present study, we show the rapid morphological changes associated with the developmental stages of bursal B cells, as well as their biological meanings although the association between large B cells and bursal development should be further investigated through overexpression (gain of function) and inhibition or removal (loss of function) of large B cells during the developmental stage.

Homeostatic proliferation (HP) is a proliferative expansion process that occurs when the absolute number of certain cells is too low to maintain at the normal level^23,24^. During embryonic development, very few pre-bursal B cells migrate and colonize into each bursal follicle. It is likely that the lymphopenic bursal environment at early time points might trigger a strong proliferation of B cells. We observed high proliferative activity and significant proliferation of bursal B cells at E17, indicating that HP-like phenomenon takes place during colonization and the subsequent expansion of B cells. Additionally, the population of B cells in bursa, but not spleen or bone marrow (Supplementary Fig. S5), is clearly divided into two subpopulations, small and large. We further demonstrated that large B cells are the major source responsible for the rapid expansion of B cell development in chickens. Therefore, it could be assumed that large B cells have a critical role in the formation of the bursal B lymphocyte pool at early time point following colonization.

MHC class II molecules, which are mainly expressed on antigen-presenting cells including B cells, present epitopes derived from extracellular proteins to helper T cells^25^. We observed that surface MHC class II appeared on almost all bursal B cells at the earliest embryonic (E) period examined (i.e., E15), but the majority of bursal B cells became MHC class II-negative with the passage of time. The extent of MHC class II expression in bursal B cells after hatching is unclear. Paramithiotis *et al*. suggested that almost all bursal B cells are MHC class II positive^26^. However, other studies have reported that MHC class II molecules are expressed only in cortical B cells^4^. Moreover, it is also known that during the bursal development, the medullary anlage first emerges on around E10–13 with entering blood-borne hematopoietic cells including MHC class II^+^ reticular cells into bursal epithelium^4^ and then MHC class II^+^ cells formed in the cortex at around late embryonic stage^27^. Considering this developmental process, MHC class II^+^ B cells present in the medulla are likely to migrate into cortical and MHC class II^+^ B cells no longer existed in the medulla, suggesting many B cells in the bursa are observed without MHC class II expression. Our results further suggested that changes of MHC class II expression are highly dependent on the developmental process, as overall expression reduced dramatically at hatching.

We have shown that IgM-positive B cells increased to more than 90% from the early developmental stage to hatching, suggesting that bursal B cells mature during embryonic development. It is intriguing that while approximately 80% of bursal B cells do not express surface MHC class II after hatching, the majority of splenic B cells continue to express MHC class II (data not shown). After hatching, approximately 5% of IgM-expressing bursal B cells migrate to the spleen^2,5^. Exactly which B cells emigrate from the bursa to the periphery is not well understood in terms of MHC class II expression. It is likely that bursal B cells would acquire MHC class II expression even after the emigration and sustain B lymphocyte pool throughout self-renewal because medullary B cells do not express MHC class II while both medullary and cortical B cells migrate to the periphery^4^.

The CD40-CD40L interaction is important in both T cell- and B cell-responses. In particular, CD40L expressed on T cells provide B cells with various effector functions including proliferation, affinity maturation, and immunoglobulin-class switching via the co-stimulatory molecule, CD40 on the B cell surface^28,29^. CD40-deficient mice showed impaired normal B cell responses^30^. Both the surface and soluble forms of CD40L affecting B cells are also expressed on mouse B cells^31^. The soluble form of CD40L has a crucial role in B cell activation and the substantial proliferation in germinal centers^29^. CD40L is also expressed on chicken B cells, and CD40/CD40L signaling is critical for the proliferation of B cells and germinal-center response in chickens^4^. Kothlow *et al*. demonstrated that, *in vitro*, CD40L stimulation promoted the survival and proliferation of chicken B cells^32^. We have observed that large B cells in the chicken bursa had higher mRNA expression of both *CD40* and *CD40L* relative to small B cells, suggesting that large B cells are mainly responsible for the rapid proliferation during bursal B cell development in chickens. Whether CD40-CD40L signaling has important functions during developmental stages of B cells in chicken bursa should be further investigated.

It is important to note that gene targeting strategy using bursal B cell line, DT40 has provided a useful tool for the loss-of-function studies during B cell development. For instance, *Ikaros* family studies with DT40 have revealed that lack of *Ikaros* exhibited the impaired B cell receptor (BCR), suggest a critical regulatory role of *Ikaros* on BCR signaling^33,34^. In addition, *Aiolos*-deficient DT40 showed a defect of activation-induced cytidine deaminase (AID) essential for the immunoglobulin gene conversion^35^. The impairment of *Helios* in DT40 led to hyper BCR signaling which is the opposite to the impact of *Ikaros*^36^. In the present study, we compared the gene expression between small and large bursal B cells based on the proliferation and differentiation of B cells. In further study, it is necessary to validate level and function of each gene at protein level in the aspect of small and large bursal B cells. E2A is associated with somatic hypermutation (SHM) of bursal B cells and the inactivation of the E2A gene leads to the abolishment of IgL chain mutations in DT40^9,37^. Considering that SHM occurs from the time of hatching, the absolute level of E2A expression at the E20 stage seems to be higher than at E18 stage although large cells have higher E2A expression than small cells. We believe it would be necessary to validate our findings with more sophisticated approaches including single cell analysis at the level of not only genomics but also transcriptomics, proteomics and metabolomics in upcoming studies.

Ig gene diversification is critical for B cell function^35^. In mammal B cells, the development of various antibody repertoires depends on rearrangement of Ig heavy and light chain having multiple functional V, D, and J gene segments^4,38^. However, antibody diversity by the Ig genes rearrangement in chicken is very limited because chickens have only a single functional V and J gene segments at Ig heavy and light chain loci^4,9,39^. Therefore, somatic gene conversion is essential for chicken B cells to generate antibody diversity. It has been suggested that gene conversion of the B cells in chicken bursa is initiated around E15 and, after hatching, it is mainly observed in the cortical area^9,38^. The cortex consists of densely packed, rapidly proliferating B lymphocytes whereas the medullar contains loosely packed lymphocytes that divide very slowly^1,9^. According to our results (Fig. 3a,b), large B cells are likely to resemble cortical cells with active gene conversion superior to small B cells in bursa. Meanwhile, *Aiolos* plays a critical role in gene conversion where Aiolos-deficient DT40 B cell showed substantial reduction of Ig gene conversion^35^. In our results (Fig. 5q), large B cell-dominant stage at E18, showed an active gene conversion with higher mRNA level of *Aiolos* than that of small B cell-dominant stage at E20. Taken together, it is likely that large cells have a higher gene conversion activity than that of small bursal B cells.

It has been suggested that the structural and functional differences of cortical and medullary cells are based on their exposure to antigen. Following BSA administration into the cloaca, high level of the antigen remains in the bursal medulla whereas undetectable in the cortical compartment^40^. Moreover, antigens derived from the blood are quickly localized to the bursal medulla whereas they are not found to the same extent in the follicular cortex^40,41^. It has been shown that most IgY^+^ cells are distributed in the medulla where antigenic stimulation, derived from the bursal lumen, play an essential role for the development of these cells^10^. Thus, considering small B cells resemble the function of medullary cells, it is probable that small B cells are related to the selection by antigen prior to the emigration to periphery.

During the early stage of B cell development in mammals, pro-B cells become pre-B cells as they express pre-BCR molecules, consisting μ heavy and surrogate light chains, on the cell surface^42^. Mammalian pre-B cells are separated into two stages, small and large pre-B cells, and each stage has phenotypical and functional differences. The proportion of large to small pre-B cells is between 1:3 and 1:4 in mice^43^. Our findings show that the ratio of large to small B cells in the chicken bursa reversed during development, changing from 2:1 at E15 to 1:4 at D4. Importantly, we demonstrated that large B cells have the dual functions of proliferation and differentiation when compared with small B cells in the chicken bursa. Future studies are needed to clarify whether large B cells divide or differentiate into small B cells or if they have different progenitors.

Because *in vitro* stimulation of mouse B cell precursors induces lymphoblasts^44^, we tested to see if large B cells could be formed by activation. *In vitro* cultures of bursal B cells with B-cell mitogen lipopolysaccharide (LPS) or phorbol 12-myristate 13-acetate (PMA) together with ionomycin did not induce proliferation and/or differentiation of large B cells (data not shown). The bursa of Fabricius, a gut-associated lymphoid tissue (GALT), is connected by a duct to the cloaca and affected by intestinal bacteria^27^. The blocking of gut-derived antigens by bursal duct ligation (BDL) suppresses B cell development^10^. We found that removal of the majority of microbiota by antibiotic treatment significantly reduced total bursal B cells, but did not affect the proportion of small to large B cells (Supplementary Fig. S6), suggesting that the gut-derived pathogen-associated molecular pattern (PAMP) signal does not have a direct effect on small and large B cells. Thus, the proportional change in small and large B cells is likely an antigen-independent, inherent feature of bursal B cells during chicken embryonic development.

Collectively, the current study defines a fundamental role of large B cells in the bursa in rapid proliferation and differentiation during B cell development. Furthermore, the proportion of small and large B cells is reversed during development, indicating a status change in bursal B cells. We suggest that a new classification system based on cell-size and granularity would be a useful indicator for bursal B cell status and stages during chicken B cell development.

## Materials and Methods

### Experimental animals and animal care

Fertile eggs from White Leghorn chickens, obtained from GBST (Green-Bio Science and Technology, Seoul National University, Pyeongchang, Korea), were used. Fertile eggs were kept at 37.5–38 °C in an incubator (Autoelex, Gimhae, Korea). The hatched young chicks were housed together under conventional conditions and were allowed free access to food and water. For antibiotic treated experiments, newly-hatched chicks were treated for 1 week with ampicillin (100 mg/l), gentamycin (100 mg/l), metronidazole (100 mg/l), neomycin (100 mg/l) and vancomycin (50 mg/l) in their drinking water. The chicks were maintained on antibiotics throughout the duration of the experiment. All experimental procedures using fertilized eggs and chickens were performed under the approval of the Institutional Animal Care and Use Committee of Seoul National University (IACUC No., SNU-150327-2). All chickens were maintained and handled in a humanely manner according to a standard management program at the University Animal Farm.

### Reagents and chemicals

Anti-chicken variants of Bu-1-FITC (clone AV20, Cat: 8395-02), Bu-1-Alexa Fluor® 647 (clone AV20, Cat: 8395-31), CD45-SPRD (clone LT40, Cat: 8270-13), MHC Class II-UNLB (clone 2G11, Cat: 8350-01), MHC Class II-FITC (clone 2G11, Cat: 8350-02), IgY-PE (clone G-1, Cat: 8320-09), IgM-SPRD (clone M-1, Cat: 8310-13), IgA-BIOT (clone A-1, Cat: 8330-08), and mouse IgG_1_-FITC (clone SB77e, Cat: 1144-02), mouse IgG_1_-PE (clone 15H6, Cat: 0102-09), mouse IgG_2b_-SPRD (clone A-1, Cat: 0104-13), and mouse IgG_2b_-BIOT (clone A-1, Cat: 0104-08) antibodies were purchased from Southern Biotechnology (Birmingham, AL, USA). Brilliant Violet (BV) 605 streptavidin (Cat: 405229) was purchased from BioLegend (San Diego, CA, USA). 7-AAD-PerCP-Cy5.5 was purchased from eBiosciences (San Diego, CA, USA). TRIzol was obtained from Ambion (Austin, TX, USA). RPMI 1640 medium, fetal bovine serum (FBS), phosphate buffered saline (PBS), and ammonium-chloride-potassium (ACK) lysing buffer were purchased from Gibco (Grand Island, NY, USA). Propidium iodide solution, chloroform, isopropanol, ethanol, diethyl pyrocarbonate (DEPC)-treated water, ethylenediaminetetraacetic acid (EDTA), hematoxylin, triton X-100, sodium citrate and bovine serum albumin (BSA) were purchased from Sigma-Aldrich (St Louis, MO, USA). Eosin was obtained from BBC Biochemical (Stanwood, WA, USA). Hoechst was purchased from Invitrogen (Grand Island, NY, USA). Endogenous peroxidase blocking solution was purchased from DakoCytomation (Glostrup, Denmark). MACS buffer was prepared with PBS containing 0.5% BSA and 2 mM EDTA. All antibiotics (ampicillin, gentamicin, metronidazole, neomycin, and vancomycin) were purchased from Sigma-Aldrich (St. Louis, MO, USA).

### Single-cell isolation

Bursa of Fabricius and spleens were collected, minced, and filtered through a 70-μm nylon cell strainer (BD Falcon, San Jose, CA, USA) to obtain a single-cell suspension. To obtain bone marrow cells, femurs and tibias were obtained after the removal of the surrounding muscles using sterile instrument. Then, both ends of the bone were cut with scissors and the marrow was flushed out with RPMI-1640 media supplemented with 5% heat-inactivated FBS using a 10-ml syringe equipped with 26-gauge (0.45-mm diameter) needle. Clusters within the marrow cell suspension were disaggregated using a 70-μm nylon cell strainer. The single bursa, spleen, and bone marrow cells were suspended in 5 ml of media and centrifuged at 300–400 × *g* for 3 min at 4 °C. Then, the pellet was treated with 1 ml of ACK lysing buffer, incubated for 3 min at room temperature, and centrifuged at 300–400 × *g* for 3 min at 4 °C. The pellet was washed and resuspended with media and filtered through a 70-μm strainer. Absolute number of the target cell population was calculated by multiplying the percentage of target cell with the total cell number of each organ examined.

### RNA extraction and cDNA synthesis

Total RNA was extracted from the bursal cells using TRIzol according to the manufacturer (Thermo Fisher Scientific)’s instruction. Briefly, single cells produced from the bursa of Fabricius were counted and treated with 1 ml of TRIzol per 1–5 × 10^6^ cells. Total RNA was isolated with the addition of 200 µl of chloroform followed by centrifugation at 12,000 × *g* for 15 min at 4 °C. The aqueous phase was transferred into a new tube and 500 µl of isopropanol was added. Then, the samples were incubated for 10 min at room temperature for RNA precipitation and centrifuged at 12,000 × *g* for 10 min at 4 °C. The RNA pellet was obtained after washing with 75% ethanol, air drying for 5–10 min, and then it was resuspended with DEPC water. RNA concentration was quantified using a NanoDrop (Amersham Biosciences, Piscataway, NJ, USA) at 260 nm. Subsequently, 500 ng of purified RNA was reverse-transcribed to cDNA using M-MLV Reverse Transcriptase (Invitrogen, Carlsbad, CA, USA) according to the manufacturer’s instructions.

### Quantitative real-time PCR

Quantitative real-time PCR was performed on cDNA using a StepOnePlus real-time PCR system (Applied Biosystems, Foster City, CA, USA). SYBR® Green PCR Master Mix (Applied Biosystems) was used according to the manufacturer’s specifications. The PCR reaction was carried out in a 96-well reaction plate with 10 μl of SYBR® green PCR master mix, 0.5 μl of primers, 1–2 μl of cDNA template, and 7–8 μl of nuclease-free H_2_O. Each reaction involved a pre-incubation at 95 °C for 10 min, followed by 45 thermal cycles at 95 °C for 15 s, 55 °C for 30 s, and 72 °C for 30 s. Relative quantification of target genes was calculated using the 2^−ΔΔCt^ method. Target-gene expression was normalized to GAPDH mRNA levels. Primers were designed using NCBI Primer-BLAST and synthesized by Bioneer Inc. (Bioneer, Daejeon, Korea), and their specific sequences are shown in Table 1.

**Table 1:** Primer sequences used for real-time quantitative PCR.

Target gene	Primer sequence	Product size (bp)
*BAFF*	F*: CACGTCATCCAGCAGAAGGAT R^#^: ACAAGAGGACAGGAGCATTGC	120
*BAFF-R*	F: CCTGGCCCCACCATAAGG R: CATTACAGTCTCTCCTCACCCATACA	120
*IL-7*	F: TCTTTCGGGTTCTGCCACTT R: CGATGTCATGGCTGAGTATGTTC	120
*IL-7R*	F: AGATGGTGATGGGACCTTTGG R: CGATGTCATGGCTGAGTATGTTC	120
*CD40*	F: TGCACACCCTGTGAGAATGGT R: CGTTGCGTTTCCATGTCTCTT	120
*CD40L*	F: TGAAGTGGATGACGACGAGCTA R: TGGTGCAGAAGCTGACTTGTG	120
*CXCL12*	F: ATCCCAAGCTAAAATGGATCCA R: AGCCCTTAACGTTCTACCCTTGA	120
*CXCR4*	F: CACAGAAGCCCTTGCGTTCT R: AGGCTTGATCCTCTGCTAACAGA	120
*RAG1*	F: CTGGTAACCCCAGTGAAATCCT R: GTGGTTAGAGAAGTGTTGGCCATA	120
*RGA2*	F: GTTGATTTTGAGTTTGGATGCTGTA R: AAGTGAATGGCCTCCCAGAA	120
*FOXO1*	F: AACCTGGCCTCCTTTTCGA R: TCAGAGCAGTGAAGCGTTGAA	120
*FOXO3*	F: TGCGTACGCGCAAGGA R: CCACACAAATCGCCTCTCTCT	120
*PAX5*	F: CCACACCCAAAGTTGTCGAA R: TGGGCACGGTGTCGTTATC	120
*BLNK*	F: CAAGCCGTGCTCTGGGTACT R: CTGTTTTCCGATCGCAGGTT	120
*EBF*	F: GGATCAGGACGGAACAGGATT R: TTTCGTGCGTGAGCAGAACT	120
*E2A*	F: GGATCAGGACGGAACAGGATT R: CAGTTTGGTCTGTGGTTTTTCG	120
*Ikaros*	F: GTGCTCATGGTTCACAATCGAA R: TTCACCCGAGTGCAACTTGA	120
*Aiolos*	F: AGACACCTGGGCTCCTACGA R: ACCGGTGTGGCTACGCTTAT	120
*Helios*	F: AGGATGCCAAGGCTTTGGAT R: GAACGCGACAGTGCTCACA	120
*GAPDH*	F: AAGGGTGGTGCTAAGCGTGTT R: GCACGATGCATTGCTGACA	120

F*, Forward primers; R^#^, Reverse primers.

### Flow cytometry analysis

Single-cell suspension of bursa, spleen, and bone marrow were stained with fluorochrome-conjugated monoclonal antibodies for 20 min at 4 °C in the dark. The primary antibodies used were the anti-chicken Bu-1-Alexa Fluor® 647, CD45-SPRD, MHC Class II-FITC, IgY-PE, IgM-SPRD, and IgA-BIOT antibodies followed by BV605 streptavidin. After staining, the cells were washed and the expression of surface markers was measured using flow cytometry (FACS Canto II, BD Biosciences). The flow cytometry data were analyzed using FlowJo software (Tree Star, San Carlos, CA, USA). Cell sorting was performed using the FACSAria sorter (BD Biosciences). Chicken B cell populations were gated on the basis of a Bu-1^+^ cell-surface phenotype and were distinguished by cell size as small (low forward- and side-scatter) or large (high forward- and side-scatter) (Supplementary Fig. S1).

### Intracellular staining

After the cell surface staining aforementioned, the cells on a 96-well plate were fixed and permeabilized with 50 µl of fixation/permeabilization buffer (eBiosciences) and incubated for 30 min at room temperature in the dark then washed with 150 µl of permeabilization buffer. The cells were resuspended in 50 µl of permeabilization buffer then stained with anti-7-AAD-PerCP-Cy5.5 for 30 min at room temperature in the dark. Then, the cells were washed with 150 µl of permeabilization buffer and analyzed using flow cytometry.

### Cell cycle analysis

Bursal cells stained with anti-chicken Bu-1 antibody were washed with PBS and fixed using ice-cold 70% ethanol for at least 1 hour at 4 °C. After washing twice with PBS, the cells were stained with 50 μg/ml of propidium iodide containing 10 μg/ml of RNase A (Invitrogen, Carlsbad, CA, USA) for 30 min in the dark. The percentages of the G0/G1, S, and G2/M phases in the small and large B cells were determined using flow cytometry.

### B cell purification with magnetic beads

Bursal cells were stained with mouse anti-chicken Bu-1 antibody. After washing with MACS buffer, the cells were incubated with anti-mouse IgG microbeads (Miltenyi Biotec) for 15 min at 4 °C in the dark and centrifuged at 300 × *g* for 10 min. Then, the cell suspension was separated on a MACS MS column in the magnetic field of a MACS Separator (Miltenyi Biotec). The magnetic fraction of positively-selected cells was used in subsequent mRNA experiments.

### Hematoxylin and eosin staining, and immunohistochemistry

Bursal tissues were fixed with 4% paraformaldehyde at 4 °C for 24 h and transferred to 30% sucrose in PBS at 4 °C for 3 days. The chicken bursa sectioned longitudinally on slide glass at 10 μm using cryocut microtome 1860 (Leica, Wetzlar, Germany). The cryosections of bursa were stained with hematoxylin and eosin (H&E) for histological analysis. For immunohistochemistry, bursa cryosections were incubated with 10 mM of sodium citrate in PBS at room temperature for 1 h. Then, the cryosections were washed for three times with PBS and endogenous peroxidase blocking solution at room temperature for 5 min. Then, cryosections were incubated with 0.3% of Triton X-100 in PBS (PBST) for 40 min. After blocking with 5% bovine serum albumin in PBST at room temperature for 30 min, the cryosections were stained with anti-chicken bu-1-FITC antibody at 4 °C for 16 h. After washing for three times with PBS, the cryosections were stained with Hoechst and visualized by digital inverted fluorescence microscope (Molecular Devices, Sunnyvale, CA, USA).

### Statistical analysis

All data are expressed as mean ± standard deviation (SD). Statistical significance was analyzed by the Student’s *t*-test using Prism version 5.01 (GraphPad Software, Inc., San Diego, CA, USA). The differences were considered statistically significant at *P < 0.05, **P < 0.01, ***P < *0.001*.

## Electronic supplementary material

Supplementary Information
