# Supplementary material for: Changes in bursal B cells in chicken during embryonic development and early life after hatching
Source: Sci Rep. 2018 Nov 15;8:16905. doi: 10.1038/s41598-018-34897-4 (PMC6238004; doi:10.1038/s41598-018-34897-4)
Supplement: Supplementary file 1 — Supplementary Information [file 41598_2018_34897_MOESM1_ESM.pdf]

Supplementary Information

Changes in bursal B cells in chicken during embryonic development and early life after hatching

Kwang Hyun Ko[1,2], In Kyu Lee[1], Girak Kim[1], Min Jeong Gu[1,2], Hyun Young Kim[3], Byung-Chul Park[4], Tae Sub Park[4], Seung Hyun Han[3] and Cheol-Heui Yun[1,2,4*]

[1]Department of Agricultural Biotechnology and Research Institute for Agriculture and Life Sciences, Seoul National University, Seoul 08826, Republic of Korea

[2]Biomodulation Major and Center for Food Bioconvergence, Seoul National University, Seoul 08826, Republic of Korea

[3]Department of Oral Microbiology and Immunology, Dental Research Institute, and Brain Korea 21 Plus Program, School of Dentistry, Seoul National University, Seoul 08826, Republic of Korea

[4]Institute of Green Bio Science Technology, Seoul National University, Pyeongchang 25354, Republic of Korea.

***Correspondence:** Cheol-Heui Yun: cyun@snu.ac.kr

Supplementary Figures

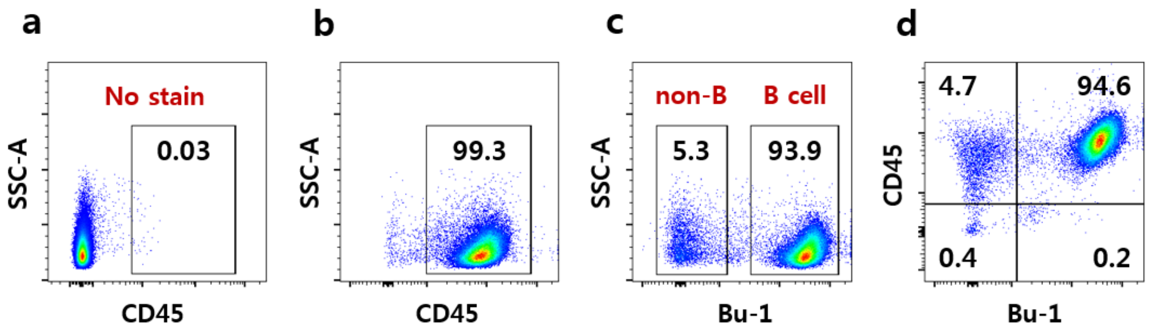

Supplementary Figure 1. Flow cytometric gating strategy for chicken B cells. To gate and analyze chicken B cells, CD45 and bu-1 antibodies were used. Single cells from bursa of Fabricius were shown (a) without stain, and stained with (b) anti-CD45, (c) anti-bu-1 or (d) both anti-CD45 and anti-bu-1 antibodies. The number in each area indicates the percentage of the cells.

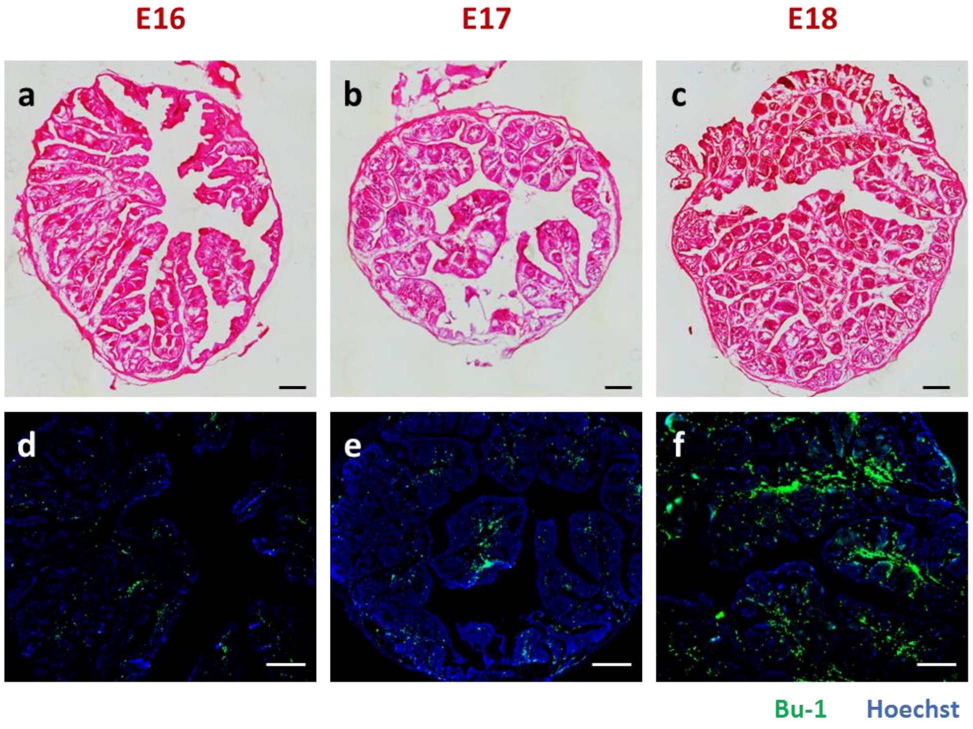

Supplementary Figure 2. Histological analysis and immunohistochemical staining of bu-1 in embryonic bursa. Bursal tissues from chicks at E16 to E18 were fixed and cryosectioned. The images for H&E staining and IHC were obtained from adjacent sections. (a-c) bursal cryosections were stained with hematoxylin and eosin (H&E) for histological analysis. (d-f) the cryosections were stained with bu-1 antibody (green) and hoechst (blue), and visualized by digital inverted fluorescence microscope. One of the representative pictures with similar result from at least 5 samples per embryonic stage is shown. Scale bars correspond to 100 μm.

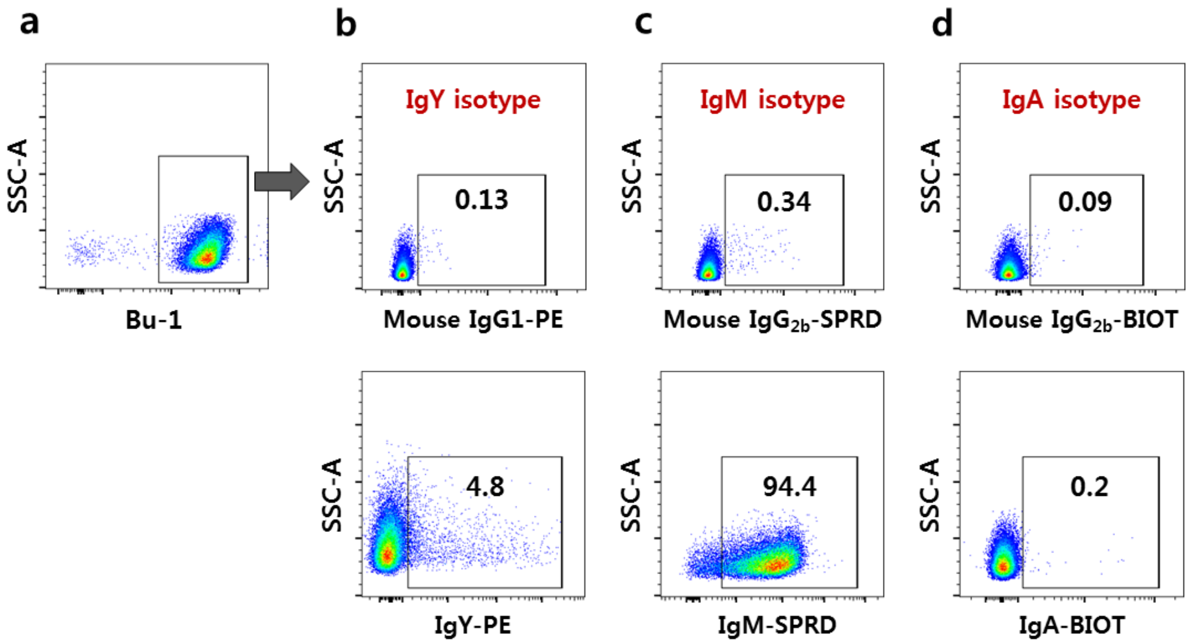

Supplementary Figure 3. Gating strategy for Ig expression on bursal B cells. Single cells produced from the bursa of 1-week-old chicks were stained with anti-bu-1 antibody or together with anti-IgY, -IgM or -IgA antibody followed by streptavidin-conjugated BV605. Then, background level was examined by using isotype control for the IgY, IgM, and IgA antibodies, respectively. (a) Bursal B cells were first identified by bu-1 gating, and then (b) IgY-, (c) IgM-, and (d) IgA-producing B cells were examined by flow cytometry. The number in each area of the panel indicates the percentage of the cells.

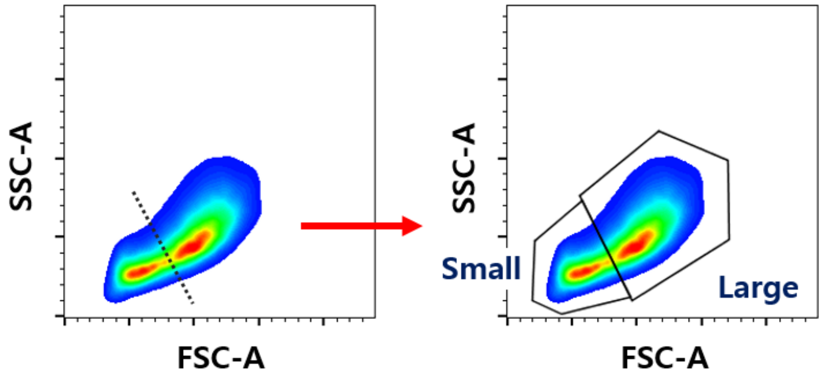

Supplementary Figure 4. Flow cytometric gating strategy for small and large B cells. Bursal cells were gated on bu-1^+ cells and distinguished by the cell size by using flow cytometry. The population gated at low forward scatter with low side scatter indicates small B cells whereas high forward with high side scatter indicates large B cells.

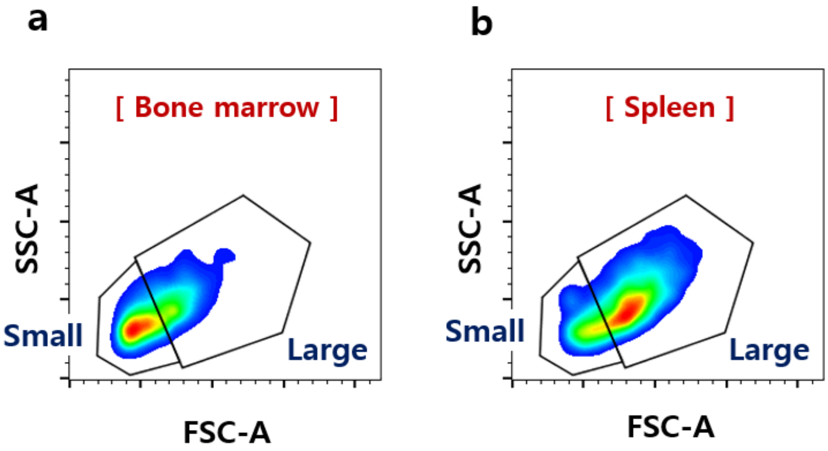

Supplementary Figure 5. Analysis of small and large B cells in the spleen and bone marrow. The single cells of (a) bone marrow and (b) spleen were gated on bu-1$^+$ cells and divided by the cell size and granularity by using flow cytometry, same as in **Supplementary Fig. 4**.

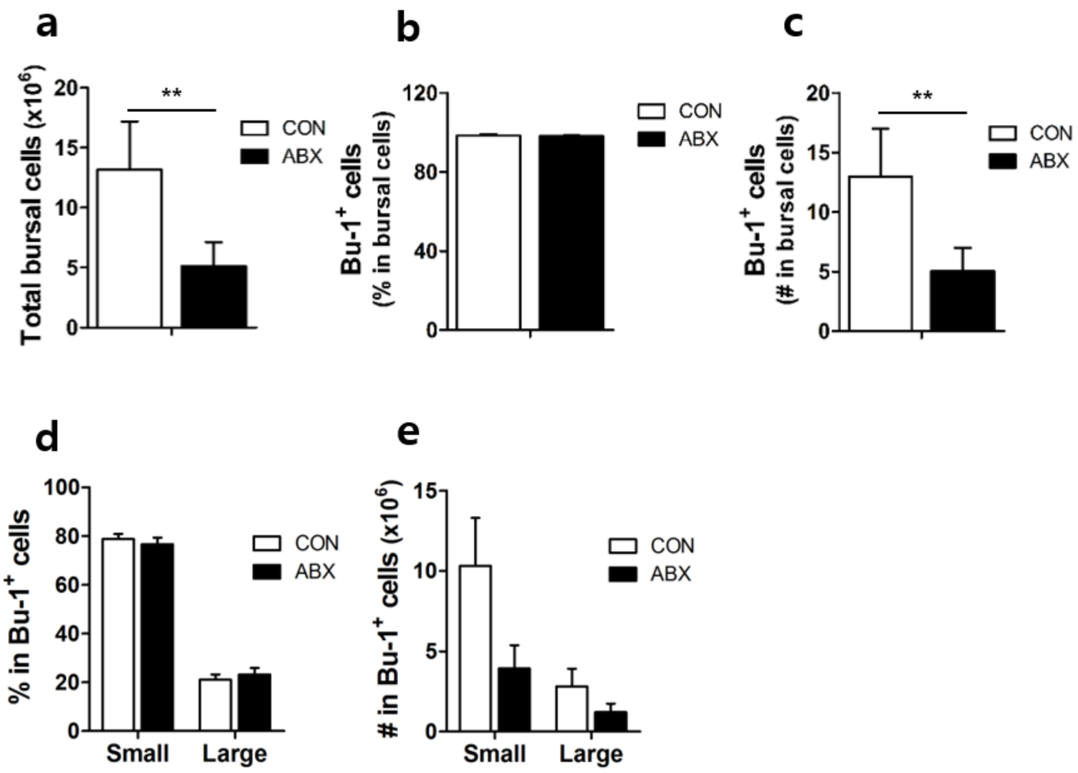

Supplementary Figure 6. Commensal microbiota affects the development of bursal B cells. Newly hatched chicks were given the combination of different antibiotics consisting ampicillin (100 mg/l), gentamycin (100 mg/l), metronidazole (100 mg/l), neomycin (100 mg/l) and vancomycin (50 mg/l) in drinking water for 1 week. Bursal cells from control (CON) or antibiotics (ABX)-treated chicks were analyzed for (a) absolute number of bursal cells, (b) percentage and (c) absolute number of bursal B cells, (d) the percentage and (e) absolute number of small and large B cells in bursa. Data represent the mean ± SD. Similar results were obtained from three separate experiments. **$P < 0.01$.
